# The Easter Holiday

**Published:** 1915-04-03

**Authors:** 


### THE EASTER HOLIDAY.

The most joyful Church festival of the year, which is also a public holiday, is undoubtedly that of Easter, for the beginning of spring generally lends to it the peculiar charm of being the first holiday of the year capable of being spent out of doors. This fact alone provides as a rule, in the concrete terms of warm weather and bright sun, the perfect outward and visible sign of that resurrection of life which is the central fact of the Church festival. Though the holiday may be short, it is more sincerely loved by many people than the formal summer holiday, which must usually be a more prosaic affair. To the outside world Christmas is the festival in which hospitals share the most, but, then, when we probe beneath the surface of the Christmas festivities we find, naturally enough, that Christmas is rather the holiday of the patients than of the hospitals. Easter does not pretend to bring to the former the same organised enjoyment; it is, from the very nature of the case, the symbol of "that drop of primeval quicksilver in the blood" which in every individual is stirring at this season of the year. Being felt also more than any other season as the turning-point of the year, it reminds us that the longest of long things and the most bitter of bitter experiences have an end; and that sorrow and suffering can no more be permanent than happiness and pleasure can be otherwise than fleeting, so that our courage is renewed and our sympathy strengthened and vivified by it. It is thus no merely smooth saying that death is swallowed up in victory, for though the one may succeed the other in individual encounters, the central fact is neither the one nor the other, but the growth and renewal of life to which death and birth, winter and spring are equally necessary. Easter, then, the Christian symbol of victory, is therefore the permanent fact, and it is perhaps our subconscious recognition of this which gives its peculiar edge of delight to the Easter holiday.

To hospital men who may tend to become hardened by familiarity with the routine of institutional administration or with the presence of suffering and disease, Easter has, or should have, a very useful lesson. For the whole nation in time of war it should have a very useful reminder. It should remind us that however essential it obviously is for us, once engaged, to prosecute the war with every effort, yet that peace and creation are the permanent things; and that war and destruction are only transitory. The hospital officer should learn from it that however important business considerations may be, the permanent thing is the spirit of helpfulness, of voluntary service, which desires simply the restoration to health of the patients, in securing which the business organisation is the means merely. Practical problems, particularly since the reception of wounded soldiers and the depletion of the medical and nursing staffs, have weighed so heavily on the shoulders of hospital managers; voluntary hospital finance is always such a pressing matter that it is well to be reminded, as Easter reminds us, even in war-time, of those ideals which have created the voluntary system, and on the continual animation of which the success of the system depends. That, rightly regarded, is the message of the Easter holiday.

